# The SAFEST review: a mixed methods systematic review of shock-absorbing flooring for fall-related injury prevention

**DOI:** 10.1186/s12877-021-02670-4

**Published:** 2022-01-06

**Authors:** Amy Drahota, Lambert M. Felix, James Raftery, Bethany E. Keenan, Chantelle C. Lachance, Dawn C. Mackey, Chris Markham, Andrew C. Laing

**Affiliations:** 1 School of Health and Care Professions, University of Portsmouth, St. Andrew’s Court, St. Michael’s Road, Portsmouth, PO1 2PR UK; 2 International Centre for Evidence in Disability, Department of Clinical Research, London School of Hygiene & Tropical Medicine, Keppel Street, London, WC1E 7HT UK; 3 Wessex Institute, University of Southampton, Alpha House, Enterprise Road, Southampton, SO16 7NS UK; 4 School of Engineering, Cardiff University, Queen’s Buildings, The Parade, Cardiff, CF24 3AA UK; 5 Department of Biomedical Physiology and Kinesiology, Simon Fraser University, 8888 University Drive East, Burnaby, British Columbia V5A 1S6 Canada; 6 Department of Kinesiology, University of Waterloo, B.C. Matthews Hall, Waterloo, Ontario N2L 3G1 Canada

**Keywords:** Accidental falls, Bone, Floors and floor coverings, Fractures, hospitals, Long-term care

## Abstract

**Background:**

Shock-absorbing flooring may minimise impact forces incurred from falls to reduce fall-related injuries; however, synthesized evidence is required to inform decision-making in hospitals and care homes.

**Methods:**

This is a Health Technology Assessment mixed methods systematic review of flooring interventions targeting older adults and staff in care settings. Our search incorporated the findings from a previous scoping review, MEDLINE, AgeLine, and Scopus (to September 2019) and other sources. Two independent reviewers selected, assessed, and extracted data from studies. We assessed risk of bias using Cochrane and Joanna Briggs Institute tools, undertook meta-analyses, and meta-aggregation.

**Results:**

20 of 22 included studies assessed our outcomes (3 Randomised Controlled Trials (RCTs); 7 observational; 5 qualitative; 5 economic), on novel floors (*N* = 12), sports floors (*N* = 5), carpet (*N* = 5), and wooden sub-floors (N = 1). Quantitative data related to 11,857 patient falls (9 studies), and 163 staff injuries (1 study). One care home-based RCT found a novel underlay produced similar injurious falls rates (high-quality evidence) and falls rates (moderate-quality evidence) to a plywood underlay with vinyl overlay and concrete sub-floors. Very low-quality evidence suggested that shock-absorbing flooring may reduce injuries in hospitals (Rate Ratio 0.55, 95% CI 0.36 to 0.84, 2 studies; 27.1% vs. 42.4%; Risk Ratio (RR) = 0.64, 95% CI 0.44 to 0.93, 2 studies) and care homes (26.4% vs. 33.0%; RR 0.80, 95% CI 0.70 to 0.91, 3 studies), without increasing falls. Economic evidence indicated that if injuries are fewer and falls not increased, then shock-absorbing flooring would be a dominant strategy. Fracture outcomes were imprecise; however, hip fractures reduced from 30 in 1000 falls on concrete to 18 in 1000 falls on wooden sub-floors (OR 0.59, 95% CI 0.45 to 0.78; one study; very low-quality evidence). Staff found moving wheeled equipment harder on shock-absorbing floors leading to workplace adaptations. Very low-quality evidence suggests staff injuries were no less frequent on rigid floors.

**Conclusion:**

Evidence favouring shock-absorbing flooring is uncertain and of very low quality. Robust research following a core outcome set is required, with attention to wider staff workplace implications.

**Trial registration:**

PROSPERO CRD42019118834.

**Supplementary Information:**

The online version contains supplementary material available at 10.1186/s12877-021-02670-4.

## Background

Addressing matters of health and safety through environmental design interventions is a long-standing and diverse discipline [1]. In geriatric care settings, one of the most prevailing safety concerns are falls and their associated injuries, the most severe of which include fractures and head injuries [2, 3]. Falls can cause lasting consequences for health, independence, functioning, and wellbeing, and are particularly discriminatory against older age groups who are more at risk of low-impact trauma and vulnerability to adverse health outcomes due to frailty [4, 5]. With a complex aetiology and array of risk factors [6–8], there remains no panacea for preventing falls and related injuries, and environmental design is therefore considered one potential tool in a repertory of interventions, including exercise and multifactorial approaches targeting individual risk factors [9].

The consensus definition of a fall being of “an unexpected event in which the participants come to rest on the ground, floor, or lower level” presents the most obvious target for environmental intervention [10]. Shock-absorbing flooring aims to reduce the stiffness of the ground surface to lower the impact forces experienced from a fall to help mitigate injury. A standard rigid floor, prevalent in care settings, may comprise a concrete sub-floor with a resilient sheet-vinyl covering (approximately 2 mm thick) [11]. Yet variations exist on this norm; commonly used floor materials such as carpet, wooden sub-floors, and thicker underlays, may variably afford softer landings for people who fall [12, 13]. In the sports sector, floor materials have been designed to offer shock-absorbency for the comfort and protection of players, and some of these designs have been repurposed for use in hospitals and care homes [14]. More recently, flooring manufacturers have started to target the gap in the market for specially-designed ‘health’ floors to support injurious falls prevention in care settings [15, 16].

Flooring interventions offer various appeals, as they form part of the ambient environment; they do not require any active user compliance, in contrast to hip protectors or helmets, which only target specific body locations. With an expected longevity of up to 20 years, a flooring intervention presents the opportunity for a significant return on investment [17–20]. If proven effective at preventing hip fractures for example (which have been estimated to incur upwards of USD 6500 per fracture to various international health systems, often considerably more [21–23]), the cost of purchasing and installing a new floor could be quickly recouped. Yet rarely do interventions come without risk, and for shock-absorbing flooring interventions the concerns are twofold: 1) the debated potential for a softer surface to inadvertently increase the risk of falling for those already unsteady on their feet [24–35]; and 2) the risk to staff who may find manoeuvring wheeled equipment (such as beds and trolleys) harder, due to greater resistance to pushing and pulling forces [36–39], potentially increasing the risk of musculoskeletal injuries [40, 41].

A scoping review identified all of the evidence on shock-absorbing flooring published to May 2016 [42]. This systematic review updated the search performed by the scoping review, and to our knowledge is the first to systematically synthesise the evidence on shock-absorbing flooring use in care settings, to help inform practice. This is an abridged report of the review, which is published in full as a Health Technology Assessment [43]. Our objectives were to:

1. Assess the potential benefits (fall-related injury prevention) and risks (falls; staff injuries) of different flooring systems in care settings;
2. Assess the extent to which these potential benefits and risks may be modified by different study/setting, intervention and participant characteristics;
3. Critically appraise and summarise current evidence on the resource use, costs and cost-effectiveness of shock-absorbing flooring in care settings for older adults, compared with standard flooring;
4. Summarise findings on the implementation of flooring interventions in the included studies;
5. Summarise the views and experiences of shock-absorbing flooring use from staff, patients’, residents’ and visitors’ perspectives;
6. Identify gaps in existing evidence.

## Methods

In this mixed methods review (including randomised, non-randomised, qualitative, and economic studies), we aimed to systematically review the evidence on shock-absorbing flooring use in care settings (hospitals and care homes) for fall-related injury prevention in older adults, to understand what is known about the effectiveness, cost-effectiveness, and qualitative experiences of shock-absorbing flooring use. We followed established approaches and reporting standards in conducting this review [44–47], which was guided by our protocol (registration: PROSPERO CRD42019118834) [48].

### Eligibility criteria

We placed no restrictions on publication status, date, or language of reports, but rather studies needed to satisfy the following characteristics:

#### Types of studies

We included primary research involving experimental, quasi-experimental, observational, and qualitative designs, and partial and full economic evaluations based on a single study or model. Laboratory/biomechanical studies, and simple before-and-after quantitative studies with no evaluation of time trends or concurrent control, were excluded.

#### Population

Our target population was broadly older adults in hospitals and care homes. Adverse events pertaining to staff were also included. Qualitative evidence evaluating the views of any individuals occupying the same environment was also eligible. Studies must have focussed on adult populations (paediatric settings were excluded); however, we did not set a strict threshold for ‘older adults’, since chronological age may not be a good indicator of frailty [49, 50], and due to the nature of the intervention we anticipated studies would have been conducted in locations where individuals were at risk of falls.

#### Settings

Studies conducted in hospitals (acute, sub-acute), intermediate and long-term care settings (nursing and care homes) were eligible. Private housing, and other settings (e.g. sporting venues) were excluded.

#### Interventions

Studies must have compared different flooring types, with at least one of the comparison groups classifiable as a ‘shock-absorbing’ floor, that is: floor coverings, underlays, and sub-floors considered to reduce the impact forces of falls. We included purposely-designed (novel) injury-prevention flooring systems, sports flooring repurposed for care setting use, carpet with or without underlay, and other combination flooring systems (e.g. vinyl overlays with padded underlays such as foam, or rubber, or wooden sub-floors). Studies involving flooring as part of a multiple-component intervention in which the effects of the floor were not discernible from the other intervention components were not eligible. Flooring is permanently affixed to the ground, providing universal coverage; therefore, fall mat interventions were excluded. Studies were eligible if they compared different types of shock-absorbing flooring systems, or a shock-absorbing floor to a standard ‘rigid’ floor (e.g. concrete, ≤2 mm vinyl/resilient sheeting). In this abridged report, we have focussed on our main comparison “any shock-absorbing flooring versus standard flooring”.

#### Outcomes

The reporting of specific outcomes did not form part of our eligibility criteria.

### Outcomes and prioritisation

Our pre-specified outcomes were developed, and prioritised, based on related core outcome sets, public involvement, wider stakeholder engagement [51], and peer review feedback on our protocol [48].

Primary outcomes:

1. Injurious falls rate per 1000 person days;
2. Falls rate per 1000 person days.

Secondary outcomes:

(3) Number of falls with injuries (e.g. none, minor, moderate, severe, death);
(4) Number of fractures;
(5) Number of hip fractures;
(6) Number of fallers;
(7) Number of adverse events (staff injuries);
(8) Number of head injuries;
(9) Fractures per 1000 person days;
(10)  Hip fractures per 1000 person days;
(11)  Qualitative outcomes (e.g. staff, patient/resident, and visitor attitudes, views, and experiences);
(12)  Economic outcomes (to include assessments of quality-adjusted life years);
(13)  Process outcomes (e.g. ease of, or problems with, flooring installation).

### Search methods

We incorporated the search results from a scoping review (which included searches from databases’ inception dates to May 2016) [42], and ran an updated search of AgeLine (EBSCO*host*), Cumulative Index of Nursing and Allied Health Literature (CINAHL; EBSCO*host*), MEDLINE (EBSCO*host*), Scopus (Elsevier), Web of Science (Thomson Reuters), and NHS Economic Evaluation Database (Centre for Reviews and Dissemination); databases were last searched in September or November 2019. Grey literature searches were conducted of conference proceedings, websites, theses, and clinical trial registries. We conducted forward and backward citation searches on included studies, and a hand search was undertaken of the journal ‘Age and Ageing’. No language restrictions were placed on the search. The MEDLINE search strategy is provided as an example in an additional file (see Additional File 1).

### Data collection

#### Data management

Search results were imported into a reference management software (Endnote™ online, Clarivate Analytics), and duplicates were removed. Screening and data extraction were supported by software (Covidence and Microsoft Excel). Data were analysed and synthesised in RevMan (version 5.3), NVivo QSR, and GRADE Pro GDT.

#### Study selection

Titles, abstracts, and full reports were screened independently by two review authors (LF plus AD, BK, CL, or OO) using an eligibility checklist. Disagreements were resolved through discussion and a third person.

#### Data extraction

For quantitative and qualitative studies, the study characteristics (funding, study design, interventions/phenomena of interest, population, outcome acquisition/methods, setting, public/patient involvement), risk of bias assessments, and outcome data, were collected independently in duplicate (AD, LF, BK, CL, KFS, CM, OO), using a data collection form and instructions (which was piloted on two studies). Disagreements were resolved through discussion and a third review author. Review authors were not involved in the assessment of primary research studies they had co-authored. Data from the economic studies were primarily collected by a health economist (JR), and checked by another review author (AD); data were collected on: research question, rationale, outcomes, perspective, time frame, costs, assumptions, and methods.

#### Risk of bias assessment and quality assessment

Studies were assessed independently in duplicate, using tools appropriate to each study design. The Cochrane Risk of Bias 2.0 tool (RoB 2.0 [52]) was used for randomised trials (with the extension for cluster trials where relevant [53]), the ROBINS-I (Risk Of Bias In Non-randomised Studies of Interventions) tool for non-randomised studies [54], and the Joanna Briggs Institute (JBI) critical appraisal checklist for qualitative studies [55]. Quantitative studies were assessed at the outcome level for our seven most prioritised outcomes (the two primary outcomes, and first five secondary outcomes). The credibility of individual findings extracted from qualitative studies were rated as ‘unequivocal’, ‘credible’, or ‘not supported’, according to the JBI criteria [55]. Problems with the quality of reporting of economic studies were assessed using the CHEERS checklist [56], and have been summarised as serious (< 50% items addressed), moderate (50 to 75% items addressed), and low (> 75% items addressed). Disagreements were resolved through discussion and involvement of a third review author if required.

### Data analysis

#### Measures of treatment effect

We summarised outcomes using rate ratios (for injurious falls rate, falls rate, fracture rate, and hip fracture rate), risk ratios (for number of falls with injuries, number of fallers, number of head injuries), or odds ratios for rare outcomes (number of fractures, number of hip fractures). We used the reported estimates and 95% confidence intervals where available, or calculated them using the raw data if feasible and appropriate. Adverse events and process outcomes were summarised narratively in text and tables.

#### Unit of analysis issues

Three studies presented potential unit of analysis issues [13, 14, 57]. We avoided the double-counting of research participants in a factorial study and multi-intervention study by segregating the data across different comparisons/sub-groups. A cluster randomised trial had presented descriptive data useful for our secondary outcomes, for which we approximated the design effect based on wider literature [58–60], using sensitivity analyses to check our assumptions.

#### Dealing with missing data

One review author (LF) approached seven corresponding authors for missing and unclear data. Missing summary effect estimates were calculated where feasible from raw data. We conducted analyses based on the available data, and assessed the problem of missing or incomplete data from individual participants as part of our risk of bias judgements.

#### Assessment of reporting bias

We did not perform any statistical tests to assess for potential reporting biases due to insufficient studies; rather, we aimed to reduce the risk of publication bias affecting our review through a comprehensive search and communication with researchers in the field. We contacted corresponding authors for missing outcomes where we thought the data may have been feasibly collected. We assessed the potential for reporting bias as part of our GRADE assessments, and downgraded the quality of the evidence if reviewers agreed it was appropriate.

### Data synthesis

Quantitative studies were synthesised in RevMan (version 5.3), with data presented separately for randomised trials and all studies combined. We pooled data using the generic inverse-variance method with a DerSimonian and Laird random effects model (assuming that intervention effects are likely to vary across studies), unless the outcome was rare, in which case we used Mantel-Haenszel analyses (fixed effect). Qualitative studies were synthesised in NVivo (version 12) using meta-aggregation to organise individual study findings into categories of similar meanings, which we subsequently pooled into synthesised findings. Economic studies were summarised narratively.

#### Assessment of heterogeneity

Heterogeneity was assessed through a combination of visual inspection of forest plots, along with consideration of tests for homogeneity (χ2 with statistical significance set at *P* < 0.10), and measures for inconsistency (I^2^) and heterogeneity (*τ* ^2^). Where feasible, we explored study design, setting, and flooring type via pre-specified subgroup analyses.

#### Sensitivity analyses

We undertook sensitivity analyses to explore the influence of risk of bias, choice of effect estimate, adjustment for clustering, use of unreported data, choice of analysis for rare events, and analysis decisions for the handling of the factorial study.

### Confidence in cumulative evidence

We appraised the quality of the evidence across the included studies at the outcome level using GRADE for quantitative outcomes [61], and the GRADE CERQual approach for qualitative findings [62]. The final set of judgements were agreed via discussion between two review authors.

### Triangulation of methods

We undertook a convergent segregated approach to mixed methods syntheses [47]; Each type of evidence (quantitative, qualitative, and economic) was analysed separately, prior to configuring the results in our overall discussion.

### Changes from the protocol

We did not search the World Health Organisation Health Evidence Network as planned, due to a technical error with their server. Originally, we had planned to use one software (Covidence) for all of our data collection tasks; however, at the time of undertaking this review it did not support all of our needs so we supplemented it with another programme (Microsoft Excel), whilst maintaining our protocol of conducting the review processes independently in duplicate. We did not perform sub-group analyses on acuity of care as planned due to limited data. Our decisions for how to deal with a 2 × 2 factorial study and rare outcomes were not planned at the protocol stage, so we undertook sensitivity analyses to ascertain the robustness of these decisions. Our protocol used the language “patient bed-days”, however we have opted for the terminology “person bed-days” in this report, as whilst the calculation is the same, the phrase better suits both care home residents and hospital patients.

## Results

**Fig. 1:**
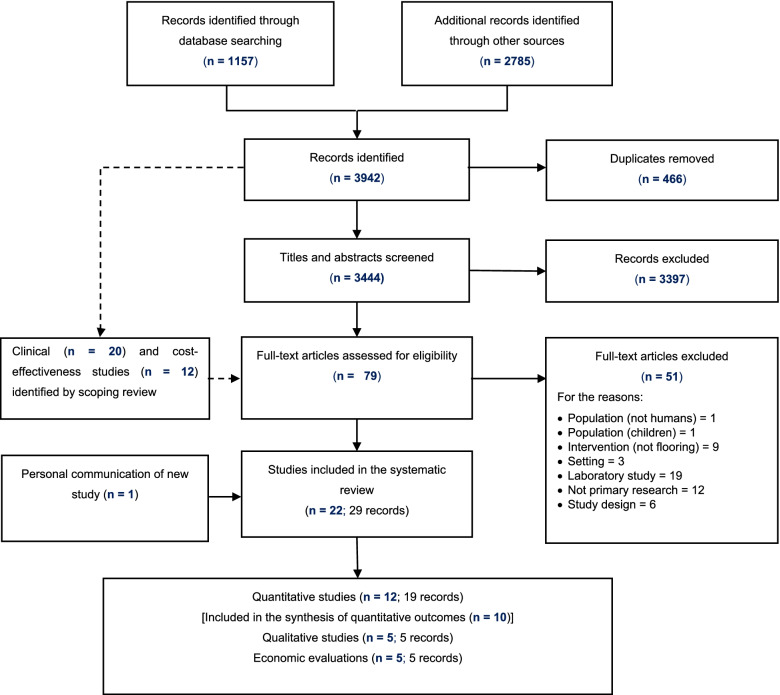
Flow diagram of study selection process

We screened 3444 records after removal of duplicates, of which 79 were assessed in full. Twenty-nine papers reporting 22 studies met our inclusion criteria (Fig. 1).

### Characteristics of included studies

We identified 12 quantitative studies [12–16, 57, 63–68], ten of which contributed data to our pre-specified outcomes (3 to 7 studies per outcome). We analysed data from three randomised controlled trials (RCTs; 1 care home-based and 2 hospital-based studies) and seven observational studies (3 care home-based and 4 hospital-based), one specific to staff injuries (163 injuries) [68], and the others relating to 11,857 patient/resident falls (11 to 6641 falls per study). The interventions explored in these were carpet (*N* = 4 studies) [12, 13, 63, 66], sports floors (Tarket Omnisports Excel, *N* = 3) [14, 57, 68], novel floors (Kradal or SmartCells, *N* = 5) [15, 16, 57, 64, 68], and wooden sub-floors (*N* = 1) [13], with three studies exploring more than one flooring type [13, 57, 68]. The flooring industry funded the costs of flooring materials and installation in three of these studies but had no further involvement in the studies’ conduct [16, 57, 68], and an additional study received a grant from the flooring manufacturer [64].

Five qualitative studies were included [69–73], representing the views and experiences of 147 people, comprising hospital/hospice staff (*N* = 84) across a range of roles (managerial, doctors, nurses, allied health professions, domestic staff), senior managers of nursing homes (*N* = 27), hospital patients (*N* = 12), hospital visitors (N = 8), residents (N = 8), and long-term care nurses (N = 8). Three qualitative studies focussed on specific flooring systems (Tarkett Omnisports Excel = 1 study; Kradal = 2 studies) [69–71], one explored perspectives towards ‘purpose-designed compliant’ flooring [72], and another was around the built environment more broadly, within which the topic of carpets was discussed [73]. None of the qualitative studies were industry-funded.

We identified five economic studies [74–78]; two studies were based in Sweden, [75, 76] and the others were based in New Zealand [77], UK [78], and the US [74]. Four studies focussed on novel flooring (3 = Kradal, 1 = dual stiffness underlay) in care homes [74–77], and one focussed on a sports floor (Tarkett Omnisports Excel on concrete) in hospitals [78]. The control floors were generally not well described, but were ‘standard’ floor coverings such as vinyl or linoleum, and in two studies the sub-floor was known to be concrete [74, 78]. The characteristics of included studies are detailed in Table 1.

**Table 1:** Characteristics of included studies

Study ID	Design / Methods	Population / Setting	Flooring systems evaluated	Outcomes
**Randomised controlled trials**				
Donald 2000 [66]	RCT	*N* = 54; Female = 44; Age = 83; Admitted for rehabilitation in a hospital, UK.	Hospital duty ‘Flotex® 200’ carpet vs. latex vinyl square tile; sub-floors NR	Incidence of falls; Injuries partially reported; Satisfaction of cleaning.
Drahota 2013 [14]	Cluster RCT	*N* = 448; Female = 353; Age = 81; NHS patients in geriatric wards across 8 hospitals, UK. Floors with a ramp test slip rating of ‘R9’ (slippery when wet).	Sports floor (8.3 mm Tarkett Omnisports Excel) vs. 2 mm vinyl (3 sites) or 2 mm thermoplastic tiles (1 site); Concrete sub-floors	Injurious falls rate; Injury severity; Fall rate; Adverse events; No. of fallers and falls.
Mackey 2019 [16]	RCT	*N* = 357; Female = 151; Age = 81.5; Residents living in study rooms of a care home, Canada.	Novel shock-absorbing floor (2.54 cm SmartCells) with 2 mm hospital-grade vinyl vs. 2.54 cm plywood with 2 mm hospital-grade vinyl; Concrete sub-floors	Serious fall-related injury; Minor fall-related injury; any fall-related injury; Falls; Fractures.
**Observational studies**				
Gustavsson 2018 [15]	Prospective cohort	*N* = 114; Female = 80; Age = 85; Residents of a care home, Sweden.	Novel shock-absorbing floor (12 mm Kradal) vs. standard vinyl/lino/ceramic; Concrete sub-floors	Injury rate per fall; Falls per 1000 PBD; No. of falls with injury; Injury severity
Hanger 2017 [57]	Prospective cohort	*N* (bedroom fallers) = 178; Female = 112; Age = 83; Admitted to a geriatric ward with a focus on medical and rehabilitation needs in a hospital, NZ.	Novel shock-absorbing floors (12 mm Kradal & 25 mm SmartCells), and a sports floor (8 mm Tarkett Omnisport Excel) vs. 3–4 mm vinyl; Concrete sub-floors	Fall rate per 1000 PBD; Fall-related injury rate per 1000 PBD; Injury severity; Injury type
Hanger 2020 [68]	Controlled cohort study	*N* (injuries) = 163; Female = NR; Age = NR; Study included any staff injury occurring on a study ward where mechanism of injury might be related to flooring in 4 older persons’ wards in a hospital, NZ.	Novel shock-absorbing floors (12 mm Kradal & 25 mm SmartCells), and a sports floor (8 mm Tarkett Omnisport Excel) vs. standard vinyl; Concrete sub-floors	Staff injuries.
Harris 2017 [65]	Prospective cohort	*N* = 36; Female = 30%; Age > 50% were 60–79 years; Assigned to one of six rooms in a telemetry unit for heart patients in a hospital; Country not clear.	Carpet tile (tufted loop with thermoplastic composite polymer backing vs. vinyl composition tile; Sub-floors NR	Preferences / satisfaction; “risk of falling” assessment ratings; behavioural mapping
Healey 1994 [12]	Retrospective cohort	*N *(falls) = 213; Female = 68; Age (median) = 85; Study involved a random sample of 225 accident forms from a care of the elderly unit over a four year period in a hospital, UK.	Carpet (varied but all single fibres rather than looped, with thin underlay) vs. vinyl; sub-floors NR	Fall related injury
Knoefel 2013 [64]	Retrospective/ Prospective cohort	*N* (falls) = 167; Female = 78; Age = 74; All documented falls on novel flooring and every 3rd fall on regular flooring.in a care home, USA.	Novel shock-absorbing flooring (SmartCells) vs. “regular floor”; sub-floors NR	No. of falls with injury; Type of injury; No. of fractures
Simpson 2004 [13]	Prospective cohort	*N* (falls) = 6641; Female = NR; Age = NR; Residents living in 34 care homes for older people, UK.	Carpet (with concrete or wooden sub-floor); Wooden sub-floor (with or without carpet); Uncarpeted (with concrete or wooden sub-floor); Concrete sub-floor (with or without carpet).	No. of falls per room; fractures per 100 falls; No. of hip fractures
Wahlström 2012 [67]	Controlled before-after study	*N* = 153; Female = 153; Age = 46; All nursing assistants; Males excluded post-hoc due to low response rate; 2 geriatric care centres, Sweden.	1.5 mm homogenous polyvinyl chloride covering with 2.5 mm foam backing (4 mm total) vs. 2 mm homogenous polyvinyl chloride covering; Concurrent control: 2.5 mm linoleum	Pain ratings in lower back, hips, knees, and feet at 6 weeks, 1 and 2 years. Adverse events not measured.
Warren 2013 [63]	Interrupted time series	*N* = 4641; Female = 2694; Age = 81; Admitted to the geriatric ward in a hospital (sub-acute), NZ.	5 mm carpet (tiles with loop pile) vs. 5 mm vinyl; concrete sub-floors.	Falls rate per 1000 PBD; fall related injuries; No. of fractures
**Qualitative studies**				
Drahota 2011 [69]	Exploring perceptions via semi-structured face-to-face interviews; Thematic content analysis	*N* = 12 patients (11 female), 8 visitors, 77 hospital staff (67 female)^a^; Study included patients/visitors in the ‘study area’, orientated to person/time/place, and staff with experience of working in the study area in 8 elderly care hospital wards, UK.	Tarkett Omnisports Excel (8.3 mm sports floor); vinyl.	The problem of falls; Protecting patients with floors; Environmental comfort; Push and pull challenges; Walking and mobilising; Cleaning and maintenance; The novelty factor; Adapting to a compliant floor; Installation.
Gustavsson 2017 [70]	Exploratory study of shared experiences; Two focus groups; Qualitative content analysis.	*N* = 8 enrolled nurses; Female = 8; Age range = 40–60; Enrolled nurses with 12 months experience of impact absorbent flooring in a nursing home, Sweden.	Kradal (12 mm closed cell tiles).	The problem of falls; Protecting patients with floors; Environmental comfort; Push and pull challenges; The novelty factor; Adapting to a compliant floor.
Gustavsson 2018 [71]	Grounded Theory study using in-depth semi-structured individual interviews.	*N* = 8 residents; Female = 6; Age range = 74–94; Study included residents with sufficient cognitive ability to participate in an interview, who lived in residential care with compliant flooring for at least 3 months; 2 nursing homes, Sweden.	Kradal (12 mm closed cell tiles).	The problem of falls; Protecting patients with floors.
Lachance 2018 [72]	Exploring perceptions via in-depth, semi-structured face-to-face interviews, analysed via a thematic framework method.	*N* = 18 senior managers; Female = 15; Age range = 37–66; In a senior management role at a Long-Term Care site in the locality; Involved in clinical and operational aspects, including implementing fall injury prevention interventions; 16 nursing homes, Canada.	Purpose-designed compliant flooring (a padded layer, generally found beneath vinyl or carpet).	Protecting patients with floors; Environmental comfort; Push and pull challenges; Walking and mobilising; Cleaning and maintenance; The novelty factor; Adapting to a compliant floor; Installation; Costs and funding.
Rigby 2012 [73]	Exploring experiences via guided tours and conversations lasting 1–6 h at each site. Extensive note-taking post visit of observations and conversations. Analysis method not described.	Hosts (*N*) = 14 nurse managers or ward sisters; 1 palliative care specialist nurse; 1 medical consultant. Plus other ‘interested staff’. Host staff all ‘older’ females; Host staff provided a guided tour, other ‘interested staff’ also joined in discussions; 7 hospices and 9 care homes in England & Australia.	Carpets and vinyl.	Push and pull challenges; Adapting to a compliant floor.
**Economic studies**				
Lange 2012 [75]	Cost utility analysis; Perspective: Societal; Model time horizon: 1 year; Life of floor: 20 years; Discount rate: 3%; Currency: 2011 SEK; Model: decision tree.	59 nursing home residents, Sweden	Kradal vs. linoleum	Costs: purchase, installation, medical costs associated with hip fracture and death, healthcare consumption; QALY loss due to hip fracture/death. ICER.
Latimer 2013 [78]	Cost utility analysis; Perspective: NHS and personal social service; Model time horizon: 15 years; Life of floor: 15 years; Discount rate: 3.5%; Currency: 2009/10 GBP; Model: decision tree. Measurement and valuation via EQ-5D supplemented by assumptions.	452 older adult hospital in-patients, UK	8.3 mm Tarkett Omnisports Excel vs. 2 mm vinyl / 2 mm thermoplastic tiles on concrete	Costs: installation, hospitalisation, falls of different severities, 3 month post-discharge resource use (hospital admissions, outpatient/healthcare visits, place of residence), mortality. QALYs associated with different types of falls. ICER.
Njogu 2008 [77]	Cost utility analysis; Perspective: NR; Model time horizon: 40 years; Life of floor: 40 years; Discount rate: NR; Currency: $ (assuming NZ $, price date NR); Model: decision tree.	Simulated care home residents, NZ	Kradal^b^ vs. traditional floor	Costs: additional purchase costs (not installation), hip fracture (inpatient and rehabilitation costs), cost of head injury and other fracture reported but not used in analysis. QALY loss due to hip fracture. ICER.
Ryen 2015 [76]	Cost utility analysis; Perspective: Societal; Model time horizon: 10 years; Life of floor: 20 years; Discount rate: 3%; Currency: SEK (price date NR); Model: Markov state.	Simulated care home residents, Sweden	Kradal vs. “standard” floor	Costs: installation, hip fracture (in- and out-patient and general practitioner costs, rehabilitation/physical therapy, transport), added life years. QALY weights for healthy and hip fracture states. ICER.
Zacker 1998 [74]	Cost-effectiveness and cost-benefit analysis; Perspective: Societal; Model time horizon: 40 years; Life of floor: 20 years; Discount rate: 5%; Currency: 1995 USD; Model: decision tree implicit.	Simulated high risk care home residents, USA	> 25 mm dual stiffness underlay vs. standard concrete floor	Costs: manufacture, installation, replacement, resident screening; Benefits: direct medical costs avoided, indirect morbidity avoided, indirect mortality avoided as a result of preventing hip fracture.

Footnotes: Both Kradal and Tarkett are branded commercial floors. Age = Mean, unless otherwise stated; EQ-5D = EuroQol 5 Dimensions (quality of life questionnaire); GBP = Great British Pounds; ICER = Incremental Cost Effectiveness Ratio; N = Number of participants unless otherwise stated; NHS = National Health Service; NR = Not Reported; NZ = New Zealand; PBD = Person Bed Days; NZ = New Zealand; QALY = Quality Adjusted Life Years; RCT = Randomised Controlled Trial; SEK = Swedish crowns; UK = United Kingdom; USA = United States of America; USD = United States Dollars

^a^ Ward staff included: Ward managers/deputy sisters (*N* = 11), doctors (*N* = 4), staff nurses (*N* = 14), nursing assistants/support workers (*N* = 11), physiotherapists/assistant/student physiotherapists (*N* = 11); occupational therapists (*N* = 5), domestic assistants (*N* = 9), other allied health professionals and staff roles (*N* = 12)

^b^ Based on references linked to in the report (not explicitly stated)

### Risk of bias and study quality

**Fig. 2:**
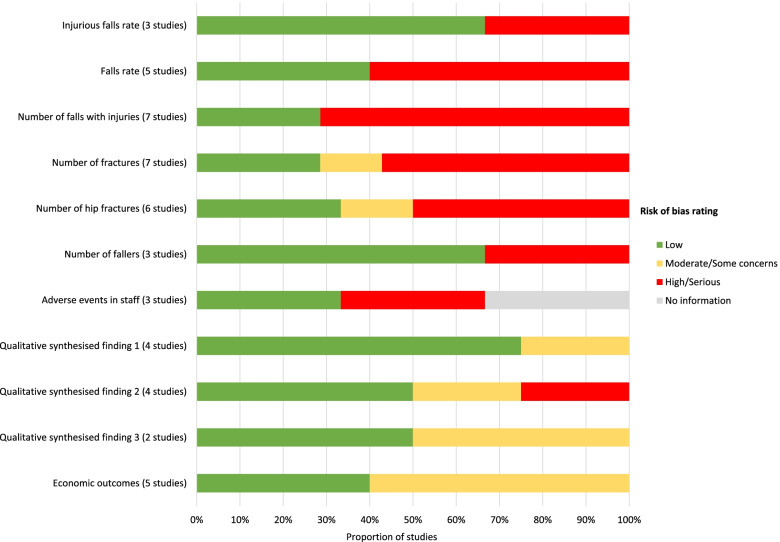
Risk of bias and quality assessments across key outcomes

A summary of our risk of bias and quality assessments is provided in Fig. 2. Of the three randomised controlled trials, two were considered at low risk of bias [14, 16], and the other presented some concerns due to lack of information on allocation concealment and reporting of injuries (which raised the risk of bias to high for number of falls with injuries) [66]. The non-randomised studies were all judged to be at serious risk of bias across all outcomes, with the predominant issue being risk of confounding.

Whilst most of the quality markers were adequately addressed in the qualitative studies, there were some issues with the quality of reporting, with none providing a detailed description of the underpinning philosophical perspective, and one lacked a clear description of the researchers’ cultural or theoretical positions [69]. One qualitative study was considered to have more serious shortcomings as it lacked a clear description of the analysis methods, and relied on personal recall to document data, meaning the report lacked representation of the participant voices [73]. This study however contributed minimal data to the synthesis, and its exclusion would not have changed the overall conclusions.

We judged there to be moderate issues in the quality of reporting of three economic studies [74, 75, 77]. More concerning than the quality of reporting, were the data used to populate the models, which varied widely. Only one, used new data from a randomised comparison [14, 78], with assumptions taken from the literature where data were very scarce [78]. Another used data from a small observational study, supplemented by best estimates from the literature made necessary by the small size of that study [75]. The other three were desktop exercises using available estimates mainly from the published literature [74, 76, 77].

### Assessment of outcomes

A summary of findings for the seven prioritised quantitative outcomes is provided separately for hospitals (Table 2) and care homes (Table 3).

**Table 2:** Summary of findings for shock-absorbing flooring versus rigid flooring in hospitals

Outcomes	Anticipated absolute effects ^**a**^ (95% CI)		Relative effect (95% CI)	Total sample size (No. of studies)	Quality of the evidence (GRADE)	Comments
	Risk with rigid flooring	Risk with shock-absorbing flooring				
**Injurious falls rate per 1000 person days**						
Randomised controlled trials	3 per 1000	**2 per 1000** (1 to 6)	**Rate ratio 0.58** (0.18 to 1.91)	9085 person days (1 RCT)	⨁⨁◯◯ LOW	These data (on sports flooring) are too imprecise to offer any certainty for this outcome.
All studies	3 per 1000	**2 per 1000** (1 to 3)	**Rate ratio 0.55** (0.36 to 0.84)	25,989 person days (2 studies)	⨁◯◯◯ VERY LOW	If 3 injurious falls a day occur in 1000 inpatients on a rigid floor, then very low-quality evidence suggests there would be one fewer injurious fall a day on a shock-absorbing floor (95% CI: 2 fewer to about the same number).
**Falls rate per 1000 person days**						
Randomised controlled trials	7 per 1000	**8 per 1000** (5 to 13)	**Rate ratio 1.07** (0.64 to 1.81)	9085 person days (1 RCT)	⨁⨁◯◯ LOW	These data (on sports flooring) are too imprecise to offer any certainty for this outcome.
All studies	7 per 1000	**6 per 1000** (5 to 8)	**Rate ratio 0.88** (0.71 to 1.09)	25,989 person days (2 studies)	⨁◯◯◯ VERY LOW	If 7 falls a day occur in 1000 inpatients on a rigid floor, then very low-quality evidence suggests that between 2 fewer falls and 1 more fall would occur a day on a shock-absorbing floor.
**Number of falls with injury**						
All studies ^b^	424 per 1000	**165 per 1000** (64 to 433)	**RR 0.39** (0.15 to 1.02)	559 falls (3 studies)	⨁◯◯◯ VERY LOW	If 424 out of 1000 inpatient falls resulted in an injury on a rigid floor, then very low-quality evidence suggests 259 fewer injurious falls would occur on a shock-absorbing floor (95% CI: 360 fewer to 9 more injurious falls). A sensitivity analysis removing a study on carpets with high risk of bias, removes the heterogeneity and increases the precision of the effect for novel/sports floors (RR = 0.64, 95% CI 0.44 to 0.93).
**Number of fractures**						
Randomised controlled trials	9 per 1000	**3 per 1000** (0 to 69)	**OR 0.33** (0.01 to 8.13)	448 participants (1 RCT)	⨁⨁◯◯ LOW	These data (on sports flooring) are too imprecise to offer any certainty for this outcome.
All studies	9 per 1000	**3 per 1000** (0 to 16)	**OR 0.28** (0.04 to 1.77)	626 participants (2 studies)	⨁◯◯◯ VERY LOW	These data (on sports and novel flooring) are too imprecise to offer any certainty for this outcome.
**Number of hip fractures**						
Randomised controlled trials	4 per 1000	**1 per 1000** (0 to 32)	**OR 0.33** (0.01 to 8.15)	448 participants (1 RCTs)	⨁⨁◯◯ LOW	These data (on sports flooring) are too imprecise to offer any certainty for this outcome.
All studies	4 per 1000	**4 per 1000** (1 to 25)	**OR 0.88** (0.12 to 6.47)	626 participants (2 studies)	⨁◯◯◯ VERY LOW	These data (on sports and novel flooring) are too imprecise to offer any certainty.
**Number of fallers**						
Randomised controlled trials	99 per 1000	**223 per 1000** (56 to 895)	**RR 2.25** (0.56 to 9.04)	502 participants (2 RCTs)	⨁◯◯◯ VERY LOW	These data (on sports flooring and carpet) are too imprecise to offer any certainty.
**Adverse events**						
Randomised controlled trials	Staff raised concerns about moving wheeled equipment on sports floor. One staff member pulled lower back on the intervention floor over 12 months follow-up.			Not reported (1 study)	⨁◯◯◯ VERY LOW	
Observational studies	No evidence to suggest higher risk of injury on intervention flooring (28 injuries in 30 months) compared to three concurrent control wards (30 injuries per ward) or a post-intervention control site (45 injuries in 30 months).			Not reported (1 study)	⨁◯◯◯ VERY LOW	

^a^The risk with shock-absorbing flooring (and its 95% confidence interval) is based on the assumed risk with standard flooring (taken from Drahota 2013 [14]) and the pooled relative effect of the intervention (and its 95% CI). ^b^ These data should be interpreted with caution as the denominator (falls) used in the calculation of RR is count data. All data contributing to this outcome are considered observational. CI: Confidence interval; OR: Odds ratio; RR: Risk ratio. Suggested definitions for grades of evidence have been published elsewhere [79]

**Table 3:** Summary of findings for shock-absorbing flooring versus rigid flooring in care homes

Outcomes	Anticipated absolute effects^**a**^ (95% CI)		Relative effect (95% CI)	Total sample size (studies)	Quality of the evidence (GRADE)	Comments
	Risk with rigid flooring	Risk with shock-absorbing flooring				
**Injurious falls rate per 1000 person days**						
Randomised controlled trials	3 per 1000	**3 per 1000** (2 to 4)	**Rate ratio 0.91** (0.62 to 1.32)	213,854 person days (1 RCT)	⨁⨁⨁⨁ HIGH	This study compared a novel underlay with vinyl overlay and concrete sub-floor, to a plywood underlay with vinyl overlay and concrete sub-floor.
All studies	3 per 1000	**3 per 1000** (2 to 4)	**Rate ratio 0.91** (0.62 to 1.32)	308,981 person days (2 studies)	⨁◯◯◯ VERY LOW	Data are missing from one observational study (novel vs rigid floors), at high risk of bias which did not report on this outcome.
**Falls rate per 1000 person days**						
Randomised controlled trials	8 per 1000	**10 per 1000** (7 to 14)	**Rate ratio 1.21** (0.87 to 1.68)	213,854 person days (1 RCT)	⨁⨁⨁◯ MODERATE	This study compared a novel underlay with vinyl overlay and concrete sub-floor, to vinyl with a plywood underlay and concrete sub-floor.
All studies	8 per 1000	**7 per 1000** (4 to 13)	**Rate ratio 0.87** (0.47 to 1.62)	308,981 person days (2 studies)	⨁◯◯◯ VERY LOW	
**Number of falls with injury**						
All studies ^b^	330 per 1000	**264 per 1000** (231 to 300)	**RR 0.80** (0.70 to 0.91)	2800 falls (3 studies)	⨁◯◯◯ VERY LOW	If 330 out of 1000 resident falls resulted in injury on a rigid floor, then very low-quality evidence suggests that 66 fewer injurious falls would occur a shock-absorbing floor (95% CI: 99 fewer to 30 fewer injurious falls).
**Number of fractures**						
Randomised controlled trials	58 per 1000	**44 per 1000** (18 to 106)	**OR 0.74** (0.29 to 1.92)	357 participants (1 RCT)	⨁⨁◯◯ LOW	These data (on novel flooring versus vinyl on plywood underlay) are too imprecise to offer any certainty over this outcome.
All studies ^b^	11 per 1000	**7 per 1000** (3 to 16)	**OR 0.61** (0.26 to 1.48)	2074 falls (2 studies)	⨁◯◯◯ VERY LOW	These data are too imprecise to offer any certainty over this outcome.
**Number of hip fractures**						
Randomised controlled trials	12 per 1000	**11 per 1000** (2 to 76)	**OR 0.94** (0.13 to 6.74)	357 participants (1 RCT)	⨁⨁◯◯ LOW	These data (on novel flooring versus vinyl on plywood underlay) are too imprecise to offer any certainty over this outcome.
All studies ^b^	2 per 1000	**3 per 1000** (2 to 4)	**OR 1.17** (0.77 to 1.80)	8548 falls (2 studies)	⨁◯◯◯ VERY LOW	These data are too heterogeneous to offer any certainty over this outcome.
**Number of fallers**						
Randomised controlled trials	676 per 1000	**697 per 1000** (602 to 798)	**RR 1.03** (0.89 to 1.18)	357 participants (1 RCT)	⨁⨁⨁⨁ HIGH	This study compared a novel underlay with vinyl overlay and concrete sub-floor, to vinyl with a plywood underlay and concrete sub-floor.
**Adverse events**						
All studies	There was no evidence to suggest an increase in force-induced musculoskeletal injuries in care home staff			Not reported (1 study)	⨁◯◯◯ VERY LOW	Personal communication. Nested pre-post design in RCT study.

^a^ The risk in the intervention group (and its 95% confidence interval) is based on the assumed risk taken from the comparison group of the RCT data and the pooled relative effect of the intervention (and its 95% CI). ^b^ These data should be interpreted with caution as the denominator (falls) used in the calculation of RR is count data. All data contributing to this outcome are considered observational. CI: Confidence interval; OR: Odds ratio; RR: Risk ratio. Suggested definitions for grades of evidence have been published elsewhere [79]

#### Injurious falls rate per 1000 person days

**Fig. 3:**
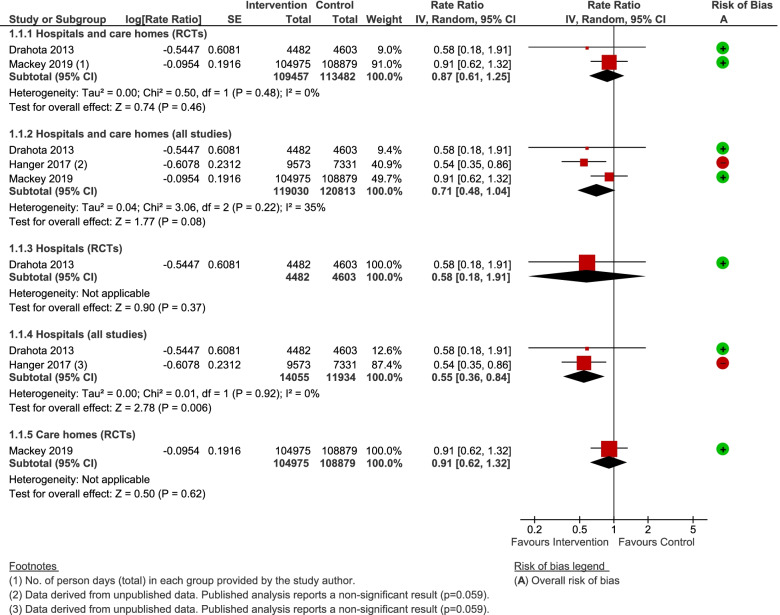
Any shock-absorbing flooring versus rigid flooring for injurious falls rate per 1000 person days

RCT evidence provided no clear evidence to support shock-absorbing flooring use for reducing injurious falls rates (Rate Ratio [RaR] = 0.87, 95% CI 0.61 to 1.25, *P* = 0.46; 2 studies; I^2^ = 0%; Fig. 3) [14, 16]. Incorporating unpublished data from an observational study [57], maintained the possibility of the intervention making no difference, albeit the effect estimate shifted more in favour of the intervention (RaR 0.71, 95% CI 0.48 to 1.04, *P* = 0.08; 3 studies; Fig. 3). There was no evidence of a differential effect by study design, setting, or flooring type (novel and sports floors). When looked at in isolation, the hospital-based evidence (RCT and unpublished observational data) was indicative of a positive effect (RaR = 0.55, 95% CI 0.36 to 0.84, *P* = 0.006; 2 studies; I^2^ = 0%; very low-quality evidence; Fig. 3). Sensitivity analyses made no material difference to the conclusions.

#### Falls rate per 1000 person days

**Fig. 4:**
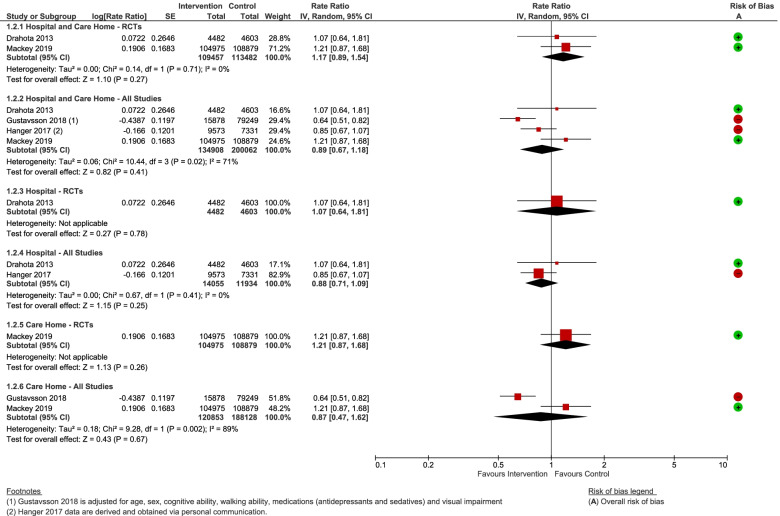
Any shock-absorbing flooring versus rigid flooring for falls rate per 1000 person days

Pooled analyses of falls rates (for RCTs alone, and all study types combined) all incorporated the possibility that shock-absorbing flooring does not affect falls rates (based on novel and sports floors). One observational study of carpet versus vinyl, unsuitable for meta-analysis, was also non-significant [63]. Heterogeneity exists amongst the studies, with the confidence intervals of RCT evidence at low risk of bias additionally incorporating the possibility that shock-absorbing flooring may increase the rate of falls (RaR = 1.17, 95% CI 0.89 to 1.54, *P* = 0.27; 2 studies; I^2^ = 0%; Fig. 4), and observational studies at high risk of bias in favour of shock-absorbing floors (test for sub-group differences: Chi^2^ = 5.44, df = 1 (*P* = 0.02); I^2^ = 81.6%). There was no evidence of a differential effect by setting, or flooring type. Sensitivity analyses did not alter the conclusions.

#### Number of falls with injuries

**Fig. 5:**
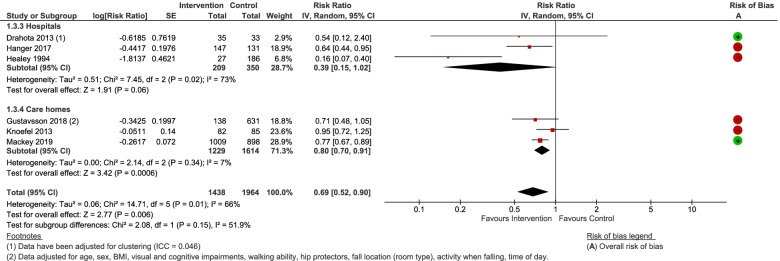
Any shock-absorbing flooring versus rigid flooring for number falls resulting in injury

For this outcome we have treated the RCT data as if they are observational, since the outcome denominator is based on the observed number of falls and not the numbers of people randomised to each group. Overall (Fig. 5), the findings positively favour shock-absorbing flooring (RR = 0.69, 95% CI 0.52 to 0.90; *P* = 0.006), but are heterogeneous (Tau^2^ = 0.06; Chi^2^ = 14.71, df = 5 (*P* = 0.01); I^2^ = 66%). This heterogeneity can be largely explained by a retrospective cohort study at serious risk of bias comparing carpet versus vinyl [12], which counter-intuitively was more favourable than the studies on novel and sports floors [14–16, 57, 64]. There was no evidence of a differential effect by setting, or study design. Novel and sports floors produced similar findings, albeit the sports floor data were very imprecise (test for subgroup differences: Chi^2^ = 0.81, df = 1 (*P* = 0.37), I^2^ = 0%), but the study on carpet introduced heterogeneity (test for subgroup differences: Chi^2^ = 12.09, df = 2 (*P* = 0.002), I^2^ = 83.5%). The hospital data (RR = 0.39, 95% CI 0.15 to 1.02, I^2^ = 73%) were sensitive to the estimated intracluster correlation coefficient (ICC) of the cluster randomised trial [14], with a smaller ICC providing a more precise effect estimate in favour of the intervention floors (RR = 0.40, 95% CI 0.16 to 0.97; *P* = 0.04; I^2^ = 73%). In addition, the removal of the carpet study from the hospital subgroup resolved the heterogeneity and provided a more precise estimate in favour of shock-absorbing floors (RR = 0.64, 95% CI 0.44 to 0.93; *P* = 0.02; I^2^ = 0%).

#### Number of fractures

**Fig. 6:**
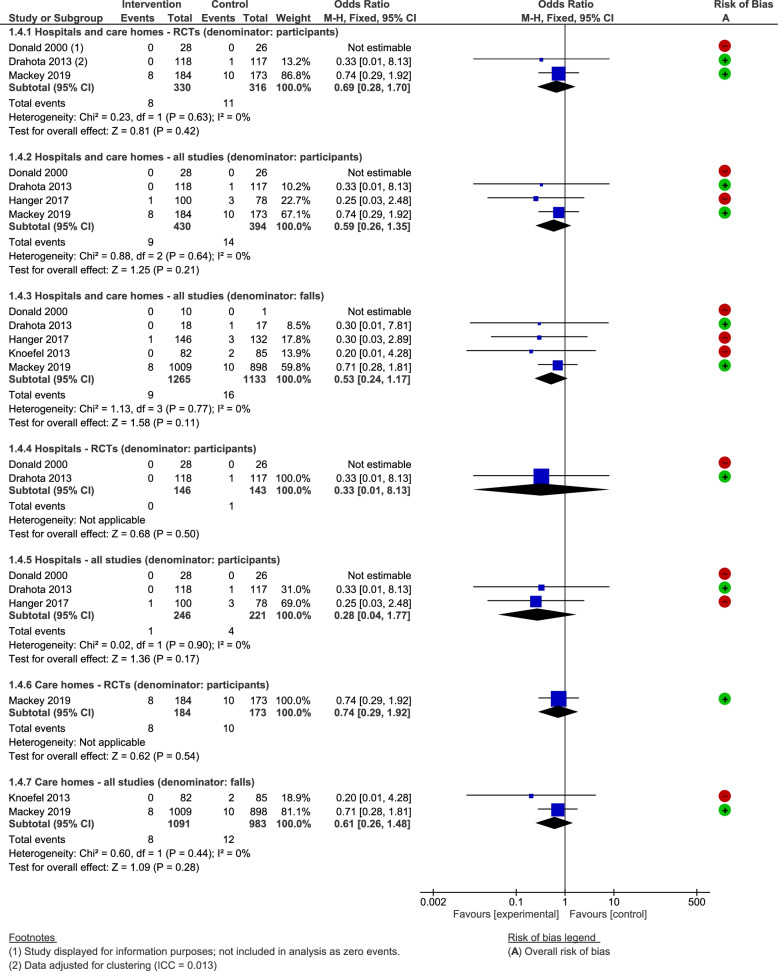
Any shock-absorbing flooring versus rigid flooring for number of fractures

Five studies reported on the outcome ‘any type of fracture’ [14, 16, 57, 64, 66]. We analysed fractures as a function of the number of participants, and the number falls to incorporate all of the evidence, however the latter may produce more biased effect estimates. The data were however too imprecise to detect with certainty whether shock-absorbing flooring reduces fracture risk (Fig. 6), as whilst the point estimates favour the intervention, the confidence intervals incorporate the possibility of no difference. We found no evidence for a differential effect between study designs, settings, or flooring types. Sensitivity analyses did not alter the bottom-line conclusions.

#### Number of hip fractures

**Fig. 7:**
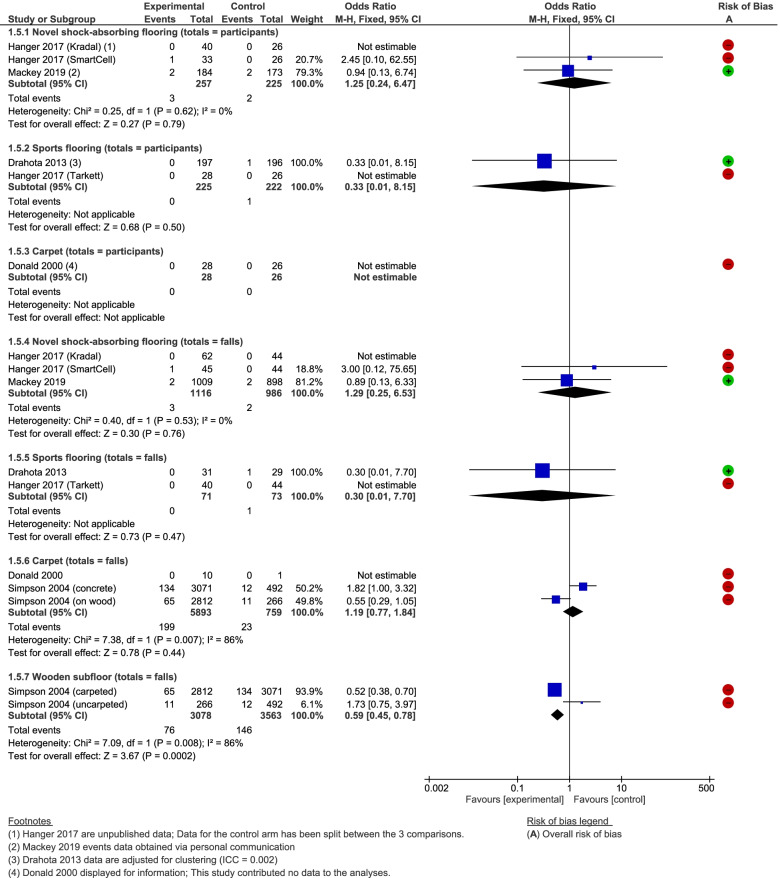
Any shock-absorbing flooring versus rigid flooring for number of hip fractures

Four studies reported hip fracture outcomes [13, 14, 57, 66]. As for the number of fractures, Fig. 7 presents hip fracture data as a function of the number of participants and falls (here we have presented the data sub-grouped into flooring types). There was insufficient evidence to detect an effect related to overlay/underlay materials in hospitals, care homes, or both settings combined, and no indication of a differential effect by study design, setting, or flooring material. The data for wooden sub-floors was indicative of a beneficial effect however (OR = 0.59, 95% CI 0.45 to 0.78 (*P* = 0.008); serious risk of bias). Assuming that 4 out of 100 falls result in a hip fracture (based on the control arm of Simpson 2004 [13]), the effect estimate can be re-expressed to suggest that one less person will fracture their hip for every 63 falls (95% CI 47 to 118 falls) that occur on wooden as opposed to concrete sub-floors (very low-quality evidence). Our sensitivity analyses did not alter the bottom line findings, however not stratifying the factorial study by the other factor in that study removes the statistical heterogeneity from all of the analyses [13].

#### Number of fallers

**Fig. 8:**
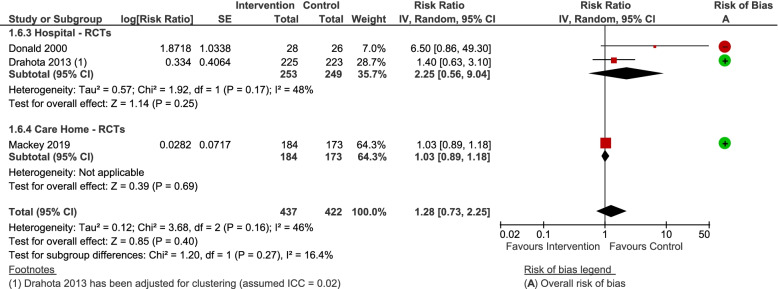
Any shock-absorbing flooring versus rigid flooring for number of fallers

Three RCTs contributed data to this outcome [14, 16, 66]. Whilst the point estimate favours control floors, the confidence intervals incorporate the possibility that shock-absorbing flooring does not increase the risk of being a faller (RR = 1.28, 95% CI 0.73 to 2.25; *P* = 0.40; I^2^ = 46%; Fig. 8). There was no indication of a differential effect by setting, or flooring type. Sensitivity analyses did not alter the bottom-line findings, although removing the study at high risk of bias (on carpet) also removed the heterogeneity and produced an effect estimate more centred around the line of no effect (RR = 1.04, 95% CI 0.90 to 1.19 (*P* = 0.60); 2 studies on novel and sports floors).

#### Number of adverse events (staff injuries)

Two RCTs collected data on staff injuries [14, 16], however since the unit of allocation in Mackey 2019 was the resident room [16], the data pertaining to staff injuries working within the same facility are more akin to a pre-post design and have not been published. One further observational study has been published [68]. Neither of the hospital-based studies were able to determine the size of the denominator population, as adverse events may have related to any events occurring on the participating wards involving staff based internally or externally to the wards, meaning accurate exposure time was not obtained. Overall, the data suggest that whilst initial concerns of working on a shock-absorbing floor maybe raised, there is very low-quality evidence to suggest that over longer periods of follow-up there may be no difference in staff injuries (Table 4).

**Table 4:** Adverse events associated with staff outcomes

Study ID	Main findings	Comments	Risk of bias
Drahota 2013 [14]	Concerns raised and 1 pulled lower back in intervention arm. No adverse events reported in control arm (12 month follow-up).	More data provided in qualitative outcomes.	Low
Hanger 2020 [68]	There were no statistically significant differences in staff injuries between intervention (28 injuries in 30 months) and concurrent control wards (average 30 injuries per ward), or with the post-intervention control ward (45 injuries in 30 months).	Quality of reporting improved post-intervention.	High
Mackey 2019 [16]	The intervention did not increase force-induced musculoskeletal injuries (24 month follow-up).	Unpublished data. Based on pre-post nested design.	Not assessed

#### Number of head injuries

**Fig. 9:**
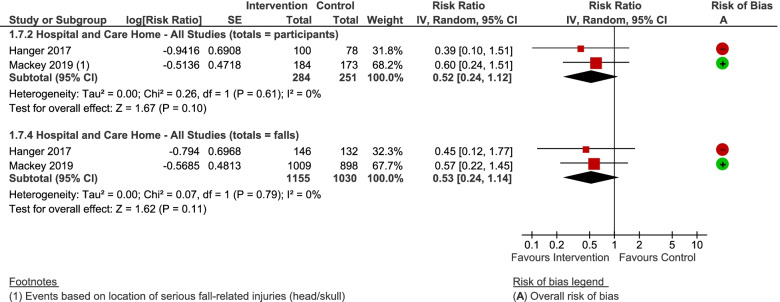
Any shock-absorbing flooring versus rigid flooring for head injuries

Two studies reported the number of head injuries [16, 57], and we also incorporated personally communicated data pertaining to Gustavsson 2018 in a sensitivity analyses [15]. We analysed the data with both participants and falls as the denominator since it was not clear whether the number of events were independent or related to recurrent fallers, however this made negligible difference to the findings (Fig. 9). Whilst the confidence intervals incorporate a reduction in the number of head injuries, the data were too imprecise, and the possibility remains that shock-absorbing flooring makes no meaningful difference, when focussing on the RCT data alone (RR = 0.60, 95% CI = 0.24 to 1.51, *P* = 0.28) or both studies combined (RR = 0.52, 95% CI 0.24 to 1.12; *P* = 0.10; I^2^ = 0%). The two studies were statistically similar, although they were conducted in different settings and using different study designs (test for subgroup differences: Chi^2^ = 0.26, df = 1 (*P* = 0.61), I^2^ = 0%); there is no indication of a differential effect due to flooring type. A sensitivity analysis including unpublished data improves the precision of the effect estimate and indicates that shock-absorbing flooring may help reduce head injuries (RR = 0.55, 95% CI 0.31 to 0.97, *P* = 0.04; 3 studies; I^2^ = 0%). These additional data were from an observational study at high risk of bias and had not been adjusted for confounding. Other sensitivity analyses did not materially affect the findings.

#### Fractures and hip fractures per 1000 person-bed days

Whilst it was possible to derive fracture and hip fracture rates for three of the studies [14, 16, 57], the analyses did not provide any further information above and beyond the data we have already reported for number of fractures/hip fractures, and are problematic due to sparseness of data leading to imprecision.

#### Qualitative findings

**Fig. 10:**
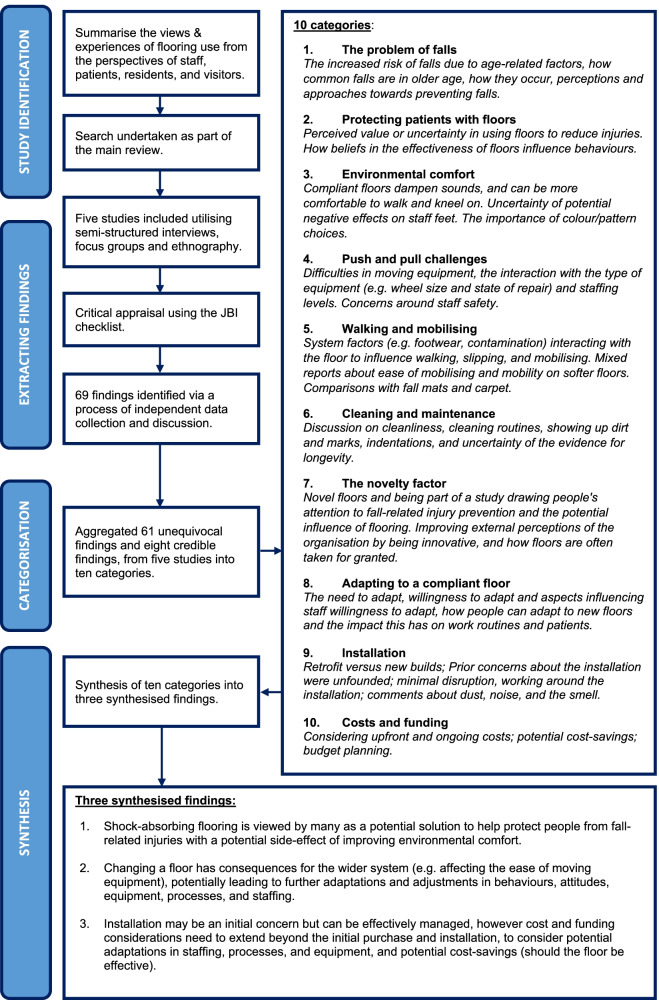
Qualitative synthesis flow chart

Five qualitative studies generated 69 findings (61 unequivocal and 8 credible), creating ten categories, which generated three synthesised findings (Fig. 10).

Qualitative finding 1: We have moderate confidence that shock-absorbing flooring is viewed by many as a potential solution to help protect people from fall-related injuries, with a potential side effect of improving environmental comfort.

Participants acknowledged that falls were a problem in older age, and that shock-absorbing flooring is a potential solution to help prevent injurious falls. Senior managers in particular expressed uncertainty around the effectiveness of floors and wanted to see further evidence; however, some staff held strong beliefs about the value of intervention floors for preventing injuries. These beliefs influenced behaviours in terms of where patients were placed, and what other injury-prevention interventions were used, and provided staff with reassurance that a ‘safety net’ was in place. Benefits were discussed in terms of improved staff morale (because they perceived that they were better able to prevent injuries), noise reduction, and some conflicted findings around improved comfort when walking and kneeling. Hospital staff, patients, and visitors found sports flooring more comfortable underfoot, whereas some care home staff debated whether the novel shock-absorbing floor they were exposed to, may be more demanding to walk on, contributing to some initial subsiding muscle soreness. Mixed views were expressed around colour and pattern choices, emphasising the need to consider the influence of a new floor on the ambient environment.

**Staff member, hospital:** *because we’ve got that floor we know it’s a special floor and we, and we put our vulnerable patients in that bay because we know if they fall and they fall on that floor then they’re gonna be protected more than they would if they fell in another bay* [69].

Qualitative finding 2: We have high confidence that changing a floor has consequences for the wider system (e.g. affecting the ease of moving equipment), potentially leading to further adaptations and adjustments in behaviours, attitudes, equipment, processes, and staffing.

This finding draws together the potential consequences (whether actual or perceived) that implementing a new shock-absorbing floor can have, related to push and pull challenges, walking and mobilising, cleaning and maintenance, and the introduction of a ‘novelty factor’ in to the environment. The perceptions people held contributed to adaptations in people’s behaviours and attitudes, for example, with regards to processes (e.g. where to place the trolley on medication rounds, where to rehabilitate patients), and staffing (e.g. rotating staff members more).

**Staff member, hospital:** *I’ve actually hurt my neck today transferring a patient using a turntable um, the patient was stood on the turntable and when I went to turn it, it wouldn’t turn at all um, and that’s not usual for a turntable and it wasn’t anything that the patient or myself or my assistant were doing, it was the floor that was stopping the turntable moving… I actually hurt my neck on it because the patient didn’t move and I did move* [69].

Qualitative finding 3: We have moderate confidence that installation may be an initial concern but can be effectively managed; however, cost and funding considerations need to extend beyond the initial purchase and installation, to consider potential adaptations in staffing/processes/equipment, and potential cost-savings from fall-related injury prevention (should the floor be effective).

This finding encapsulated discussions on the installation process, which typically went smoothly even when installation was conducted as a retrofit, which was of concern to some along with the management of thresholds. Upfront costs associated with installation have budgetary implications with funding mechanisms likely to vary by setting, context, and whether it is for a new build or retrofit. Senior managers were cognisant of potential extra costs associated with obtaining compatible equipment for use with the floor, and staff training, as well as potential cost-savings.

**Senior manager, care home:** *You’d have to look at the flooring [.*. *.] at where it’s going and then if you have to look at the motorized lifts and [.. .] different equipment to accommodate the flooring. [.. .] And training for the staff on proper body mechanics. [.. .] it’s not just how much the flooring costs* [72].

#### Economic outcomes

Five studies contributed economic data [74–78], four of which were very low quality [74–77]. Whilst there was heterogeneity between the floors, settings, and population groups assessed, the assumptions made in the poorer quality studies may have been unduly optimistic. Three of these found that shock-absorbent floors dominated standard floors in that costs were less and outcomes improved [74, 76, 77], and one estimated that shock-absorbing floors increased both costs and quality-adjusted life years (QALYs) but at a cost per increased QALY well above the accepted threshold level [75]. The QALY gains in these studies were a result of assuming relatively large QALY losses due to hip fracture. Only the higher quality study collected data on quality of life [78]. This study found reduced QALYs, albeit with reduced costs, which despite a favourable incremental cost effectiveness ratio, was noted to not likely be a result leading to implementation. The reduced QALYs in this study were based on the assumption that shock-absorbing flooring increases falls risk; a sensitivity analysis demonstrated that if shock-absorbing flooring does not increase the number of fallers yet reduces the number of injurious falls, the intervention floor would become dominant.

#### Process outcomes

Whilst no process evaluations were identified, the qualitative outcomes captured process issues around installation, maintenance and ease of use. Here we report on additional information provided by the quantitative studies. One study described a 20–30 cm split seam in the new floor attributed to welding at installation which was subsequently repaired [14]. The types of floors selected in studies influenced where they were placed as some flooring types were unsuitable for wet areas. As a minimum, floors were placed in the bedded areas, and in care homes the coverage extended to other living areas (Table 5). We were able to assess the protection offered in terms of the number of falls that occurred in the target areas in three studies, to find that upwards of 75% of all falls occurred on the intervention floors for participants assigned to the intervention group, when at least the bedded areas were covered. Two quantitative hospital-based studies reported impacts on the working environment [14, 57], citing increased effort required to move wheeled equipment. One of these studies also highlighted changes to staffing to support manual handling, with one of the four intervention sites increasing staffing from six to seven staff members during the 07:00–15:00 shift, and another site altering the shift patterns (maintaining the overall staffing levels) to increase cover on the night shift [69].

**Table 5:** Floor coverage and proportion of falls occurring on target areas with intervention

Study ID	Intervention	Areas covered by intervention flooring	Total no. of falls	% of falls on target areas
**Hospitals**				
Drahota 2013 [14, 69]	Tarkett Omnisports Excel	Hospital bays (bedded areas excluding bathrooms and corridors)	68	75%
Hanger 2017 [57]	Tarkett Omnisports Excel, Kradal, & SmartCells	Hospital bays (bedded areas excluding bathrooms and corridors)	323	86%
**Care homes**				
Mackey 2019 [16]	SmartCells	Resident rooms (living, bathroom, and closet areas) excluding common areas (dining rooms, hallways, lounges, outside areas).	Not described; only bedroom falls reported.	
Gustavsson 2018 [15]	Kradal	Resident apartments, communal dining room, corridor (excluding bathrooms and outdoor areas)	851	78%

## Discussion

The findings from the different types of evidence (quantitative, qualitative, and economic) included in this review, were largely complementary of each other and focussed on different aspects of our research question; however, there were some exceptions. The qualitative evidence suggests that many people view shock-absorbing flooring as a potential solution for reducing injurious falls, and whilst the limited robust quantitative data did not confirm this to be true, very low-quality quantitative data indicates shock-absorbing flooring may have a positive effect. We cannot discern from this whether the views held by those utilising the floors are contributing to bias in the very low-quality quantitative evidence, or if the qualitative evidence is merely reflecting some truth identified by the low-quality quantitative evidence. There were no qualitative data linked to the non-significant trial contributing the more robust quantitative evidence, which may further explain this contradiction. The qualitative data additionally highlighted that senior managers were aware of the potential for additional costs associated with shock-absorbing flooring, in relation to workplace adaptations (e.g. staffing levels, training, equipment upgrades), however to date these costs have not been considered in economic evaluations.

Systematic reviews on flooring materials are sparse, with Cochrane reviews on falls prevention [9], and hospital environments [80], excluding or not fully covering the studies on shock-absorbing flooring. There has been a scoping review of shock-absorbing flooring [42], and a review on floor finishes with a facility management focus [11]; however, these were descriptive rather than analytical, and did not incorporate a risk of bias or quality assessment, nor include all of the studies we identified. The scoping review did however incorporate a broader range of literature [42], including laboratory-based and biomechanical studies exploring impact absorption, gait, and balance [24–33, 35, 48, 81–101].

We found one high quality study indicating that a novel shock-absorbing underlay was no more effective than rigid flooring in care homes [16], and very low-quality evidence that shock-absorbing flooring may reduce fall-related injuries. Laboratory studies typically indicate the promise that shock-absorbing flooring holds in terms of impact absorption, with the same underlay that was found to produce null clinical effects in the present review [16], demonstrated to attenuate peak force by up to 33.7% to the hip [25], and 80% to the head [27], in simulated laboratory falls. This disconnect may relate to: (i) the underlying assumptions of laboratory-based research (e.g. the biofidelity of test systems, impacts simulated and assumed to be involved in injuries); (ii) co-interventions or other setting or fall characteristics that may negate the power of a clinical study to detect a change attributable to flooring; or (iii) the susceptibility of study populations to low-impact trauma such that the impact attenuation achieved remains insufficient. The clinical evidence we found in favour of shock-absorbing flooring, which aligns with the indications of laboratory evidence, was of very low quality, meaning that it is very uncertain and further research is likely to improve our understanding.

Push and pull tasks have been explored in biomechanical studies, highlighting the increased forces required to move wheeled equipment [36, 102, 103]. The biomechanical literature complements our findings, and highlights the important interactions between equipment types, flooring materials, and pushing forces required, indicating the potential for risk mitigation strategies to help prevent adverse events.

A further concern related to shock-absorbing flooring is the potential for it to lead to instability and increase falls risk [26]. We found very low-quality evidence in hospitals that the rate of falls was not increased with shock-absorbing flooring, and moderate- and high-quality evidence in care homes that falls rates and faller risk were not affected by SmartCells underlay. These findings align with biomechanical evidence (often conducted with healthy adults), which supports that individuals can maintain their balance on carpet [28, 86, 87], and novel shock-absorbing floors [25, 26, 28, 30, 104, 105]. Compliant surfaces have been contraindicated however when sensory input (such as visual cues) is affected [24, 86, 94], and stroke patients have been shown to find carpet more challenging than parquetry [31]. Other biomechanical research on hospital inpatients found no difference in their ability to perform the timed-up-and-go test on novel, sports, and rigid flooring types [105]. The current direct and indirect evidence appears promising in suggesting that falls are not adversely increased on shock-absorbing floors, however the evidence is imprecise in hospital settings, and biomechanical evidence in clinical populations is sparse.

## Conclusions

There is high-quality evidence that a novel shock-absorbing underlay produces similar injury and falls rates to a rigid plywood underlay, with vinyl overlays and concrete sub-floors in care homes. When incorporating observational studies, we found very low-quality evidence that shock-absorbing flooring may reduce the number of falls resulting in injury in care homes. There is also very low-quality evidence that shock-absorbing flooring use in hospitals may reduce injuries without increasing the rate of falls. Data on fractures and head injuries were generally too imprecise to determine effectiveness in care homes and hospitals; however, one observational study at high risk of bias indicated that fewer hip fractures were likely to occur on wooden sub-floors compared to concrete sub-floors in care homes. Including unadjusted unpublished observational data on head injuries indicated that shock-absorbing flooring might reduce head injuries; however, these data are of very low quality. Whilst some adverse events were described, there is very low-quality observational evidence that novel and sports floors do not result in more staff injuries in up to two years follow-up. The qualitative data is indicative that there may have been under-reporting of adverse events in the trial data [69]. Staff did report increased effort required to manoeuvre wheeled equipment (in both quantitative and qualitative studies), which led to changes in the workplace; it is unclear whether the lack of observed influence of shock-absorbing flooring on staff adverse events is *despite* or *because* of these workplace adaptations, or due to flaws in the studies collecting data on these outcomes.

Fall-related injuries remain a significant problem for care settings [2, 3]. The present systematic review summarises evidence associated with shock-absorbing flooring, which remains a potential solution albeit the research in favour of shock-absorbing flooring has limitations and is therefore very uncertain; this has led to the following prioritised recommendations for research:

1. To establish a clearly defined core outcome set for flooring studies, which includes recommendations for measurement, analysis, and reporting.
2. Research questions lending themselves to observational designs need to address the above core outcome set, and comprehensively deal with potential confounding. Other questions (particularly on new flooring interventions) lend themselves to pragmatic randomised controlled trials, of which there are a paucity.
3. The dearth of robust research on the effectiveness of shock-absorbing flooring in hospital settings should be addressed.
4. Studies should plan for workplace adaptations within the study design, for example through process evaluations and risk management plans to better mitigate, manage and evaluate risks to staff. Further research and innovation is also required to identify how best to adapt the workplace to shock-absorbing flooring.
5. High quality economic evidence is required that provides improved specifications of the alternatives evaluated, distinguishes falls by severity and type, specifies the processes by which reductions in types of falls are expected to improve health, uses appropriate time frames, provides greater details to enable different definitions of costs to be used, and considers the costs of workplace adaptations.
6. With the uncertainty surrounding current flooring solutions, research and innovation is required to establish the specifications for improved products to support fall-related injury prevention in care settings.

## Supplementary Information

**Additional file 1.** Search strategy for Medline.

## Data Availability

The datasets supporting the conclusions of this article are available in the full report and its additional files (published with the Health Technology Assessment [43]). Project files are available on the Open Science Framework (https://osf.io/ev6xs/). Any additional data can be obtained from the corresponding author on request.

## Abbreviations

CERQual: Confidence in the Evidence from Reviews of Qualitative research
CI: Confidence Interval
CINAHL: Cumulative Index of Nursing and Allied Health Literature
GRADE: Grading of Recommendations, Assessment, Development and Evaluations
JBI: Joanna Briggs Institute
NHS: National Health Service
NIHR: National Institute for Health Research
PPI: Patient and Public Involvement
QALY: Quality-adjusted Life Years
RCT: Randomised Controlled Trial
RoB: Risk of Bias
ROBINS-I: Risk Of Bias In Non-randomised Studies of Interventions
RaR: Rate Ratio
RR: Risk Ratio
USD: United States Dollars
