# Supplementary material for: The SAFEST review: a mixed methods systematic review of shock-absorbing flooring for fall-related injury prevention
Source: BMC Geriatr. 2022 Jan 6;22:32. doi: 10.1186/s12877-021-02670-4 (PMC8739972; doi:10.1186/s12877-021-02670-4)
Supplement: Supplementary file 1 — Additional file 1. Search strategy for Medline. [file 12877_2021_2670_MOESM1_ESM.pdf]

Additional file 1: Search strategy of Medline

1	MH "Wounds and Injuries+
2	MH "Accidental Falls/PC"
3	MH "Hip Fractures+/PC"
4	Fall#
5	Faller#
6	S1 OR S2 OR S3 OR S4 OR S5
7	MH "Aged+"
8	MH "Middle Aged"
9	Older
10	Senior#
11	elderly
12	S7 OR S8 OR S9 OR S10 OR S11
13	S6 AND S12
14	MH "Residential Facilities+"
15	MH "Long-Term Care"
16	MH "Institutionalization"
17	MH "Hospitalization"
18	MH "Subacute Care"
19	MH "Hospitals+"
20	MH "Hospital Units"
21	MH "Rehabilitation Centers"
22	MH "Inpatients"
23	MH "Geriatric Assessment"
24	("long stay" or "long term" or "acute" or "sub-acute" or "subacute" or "residential" or "hospital") N3 (care or ward# or hospital)
25	(rehabilitation or geriatric) N1 (ward# or hospital# or unit# or department#)
26	Hostel# or nursing home#
27	inpatient
28	resident#
29	institution#

30 S14 OR S15 OR S16 OR S17 OR S18 OR S19 OR S20 OR S21 OR S22 OR S23 OR S24 OR S25 OR S26 OR S27 OR S28 OR S29

31 S13 and S30

32 floor* NOT (pelvic floor OR sinus OR mouth)

33 carpet*

34 ground surface#

35 smartcell*

36 tarkett

37 softile

38 sorbashock

39 forbo

40 kradal

41 noraplan

42 MH "Floors and Floorcoverings"

43 S32 OR S33 OR S34 OR S35 OR S36 OR S37 OR S38 OR S39 OR S40 OR S41 OR S42

44 S31 AND S43

45 MH "Animals+"

46 MH "Humans"

47 S45 NOT S46

48 S44 NOT S47

49 S44 NOT S47

Limiters - Date of Publication: 2016-05-01-
